# Cytohistological and Immunohistochemical Correlation of Cutaneous Mixed Tumors: A Series of Four Cases and Review of Recent Molecular Updates

**DOI:** 10.7759/cureus.47233

**Published:** 2023-10-17

**Authors:** Ruchi Rathore, Divya Aggarwal, Nadeem Tanveer, Sonal Sharma

**Affiliations:** 1 Department of Pathology, All India Institute of Medical Sciences, New Delhi, IND; 2 Department of Pathology, All India Institute of Medical Sciences, Jodhpur, IND; 3 Department of Pathology, University College of Medical Sciences, Delhi, IND

**Keywords:** mixed tumor of the skin, hmg, ewsr1, plag1, cytology of skin tumors, appendageal tumor, benign skin adnexal tumors

## Abstract

Fine needle aspiration cytology (FNAC) is an established diagnostic modality today, but its utilization in skin tumors is limited by the fact that most of these lesions are small and easily accessible for clinicians to perform an excision biopsy. As a result, our knowledge of the cytological features of some of the uncommonly encountered skin tumors is still lacking. The aim of this study was to correlate the cytological features of cutaneous mixed tumors (CMTs) with histological and immunohistochemical findings in four cases of CMT in commonly and uncommonly encountered locations. We also review the recent updates highlighting the various genetic rearrangements and newer more specific immunohistochemical markers described so far. This was a retrospective study, and all the slides were taken from our departmental archives. Case 1 was a 25-year-old male who presented with a gradually increasing painless swelling over the right angle of the mouth of 1.5 years duration. Case 2 was a 45-year-old male with swelling on the right forearm for the last three years. Case 3 was a 35-year-old female with a forehead swelling of one year duration. Case 4 was a 55-year-old female with left cheek swelling for two years. On clinical examination, all four nodular swellings were predominantly in the skin/subcutaneous tissue. On cytology, all cases showed abundant chondromyxoid material with clusters of benign epithelial cells and a fair number of predominantly singly scattered myoepithelial cells. The diagnosis of all four cases was further confirmed on histopathology and immunohistochemistry, and the findings correlated well with cytology. The cytological features of CMT closely correlate with their histopathological and immunohistochemical features. Newer immunohistochemistry (IHC) marker pleomorphic adenoma gene 1 (PLAG1) may be helpful in making a definitive diagnosis of CMT on cytology and cell block preparation along with a good clinical correlation. Complete surgical excision is the treatment of choice, and recurrence is rare.

## Introduction

Cutaneous mixed tumors (CMTs) were earlier known as chondroid syringoma (CS), but the fifth edition of the classification of skin tumors of the World Health Organization (WHO) does not recommend using this term anymore. These tumors are rare, benign adnexal neoplasms originating from the sweat gland that account for 0.01%-0.098% of all primary skin tumors [1]. They usually present as slow-growing, painless, nodular, subcutaneous lesions of the head and neck region. Infrequently, they may also be present in other locations such as the extremities, axilla, or trunk [2]. Due to their easy accessibility and unremarkable clinical features, these tumors are often diagnosed histopathologically on excision biopsy specimens and infrequently aspirated. Our series of cases not only expands on the current knowledge of the cytology of CMTs in correlation with histopathology and immunohistochemistry but also reviews the latest literature on the molecular basis of this disease.

## Case presentation

Case 1

A 25-year-old male presented with a gradually increasing painless swelling over the right angle of the mouth for the last 1.5 years. There was a history of injury with a shaving blade while shaving before the appearance of this lesion. On examination, it was a 1 cm × 1 cm firm, mobile, non-tender, nodular swelling in the subcutaneous plane over the right angle of the mouth. The overlying skin was not pinchable. The oral cavity was unremarkable, and the buccal mucosa of the oral cavity was healthy. Based on the clinical presentation and a preceding history of trauma, a clinical diagnosis of an epidermal cyst was made, and fine needle aspiration cytology (FNAC) was performed.

Case 2

A 45-year-old male presented with a nodular swelling over the right forearm near the wrist joint for the last three years. It was painless and gradually increasing in size. There was no history of trauma or any other significant complaint. On examination, it was a 1 cm × 1 cm firm, nodular, non-tender swelling in the subcutaneous plane of the right forearm. Based on clinical findings, a differential diagnosis of ganglion/epidermal cyst was rendered by the clinicians, and the patient was sent for FNAC.

Case 3

A 35-year-old female presented with a forehead swelling of one year duration with no other complaints. It was a gradually progressive, painless, 0.8 cm × 0.8 cm swelling in the subcutaneous plane on the left side of the forehead near the hairline. A clinical diagnosis of an epidermal cyst was made based on the location, and FNAC was performed.

Case 4

**Figure 1:**
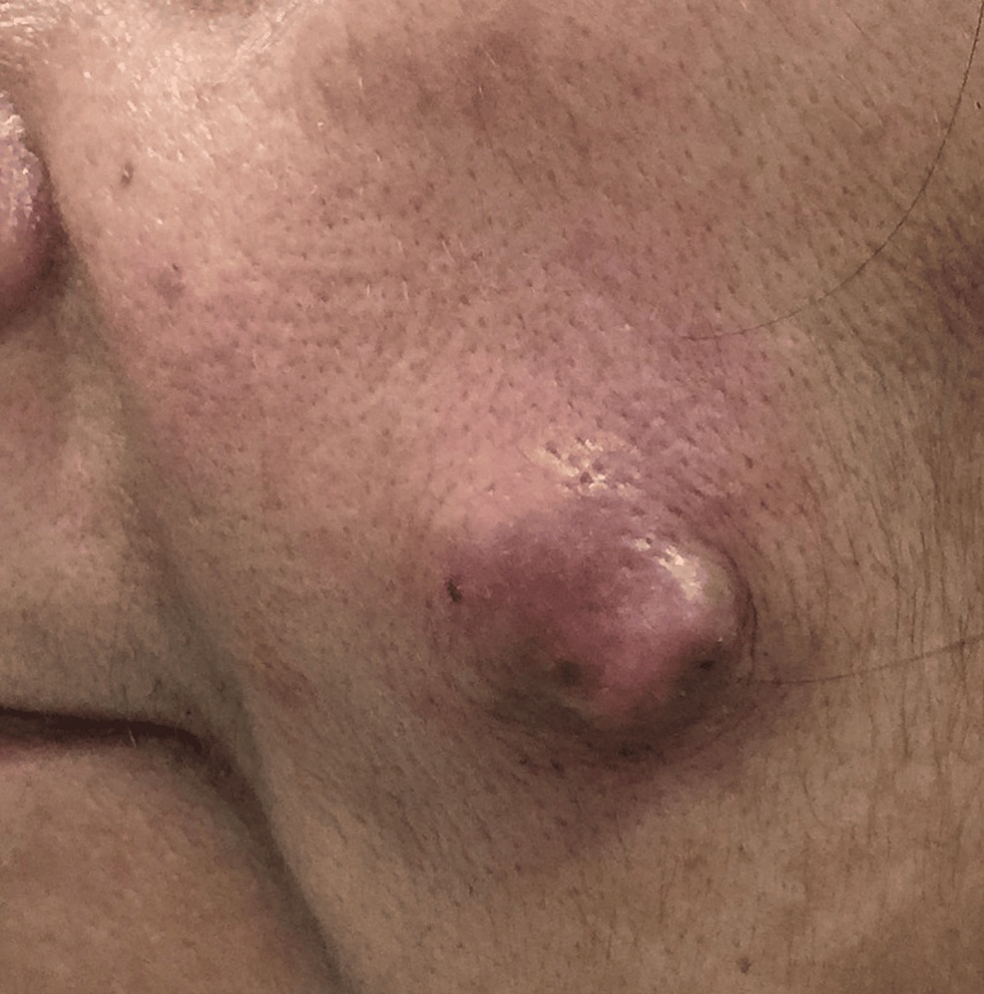
Firm, mobile swelling on the left cheek in case 4

A 55-year-old female presented with a slow-growing, painless swelling on the left cheek for two years (Figure 1). It was gradually increasing in size and was 2 cm × 2 cm on presentation. The outer surface seemed variegated; however, the oral mucosa was unremarkable. There was no history of trauma or pain in the past. On examination, it was a firm, mobile, non-tender, subcutaneous swelling where the overlying skin could not be pinched. The oral cavity examination was unremarkable. Based on history and clinical findings, a diagnosis of CMT was made, and FNAC was performed. A written consent was taken from the patient for using her image for educational and publishing purposes.

For cases 1-4, after the initial diagnosis of a cutaneous mixed tumor on FNAC, an excision biopsy was performed and sent for histopathological examination. The histopathology diagnosis was further confirmed by immunohistochemistry on formalin-fixed paraffin-embedded tissues for pancytokeratin (PanCk), S-100, vimentin, and glial fibrillary acid protein (GFAP). Table 1 presents a summary of cases 1-4.

**Table 1:** Summary of cases 1-4 EC: epidermal cyst

Case number	Age	Sex	Clinical features	Duration	Location	Clinical diagnosis	Cytological diagnosis	Histopathological diagnosis
1	25	Male	Slow-growing, painless mass	1.5 years	Right angle of the mouth	History of trauma: misdiagnosed as EC	Cutaneous mixed tumor	Cutaneous mixed tumor
2	45	Male	Nodular swelling	3 years	Right forearm	Near the wrist joint: misdiagnosed as ganglion/EC	-do-	-do-
3	35	Female	Slow-growing, painless mass	1 year	Forehead	Near the hairline: misdiagnosed as EC	-do-	-do-
4	55	Female	Slow-growing, painless mass	2 years	Left cheek	Diagnosed as cutaneous mixed tumor	-do-	-do-

Cytological features

**Figure 2:**
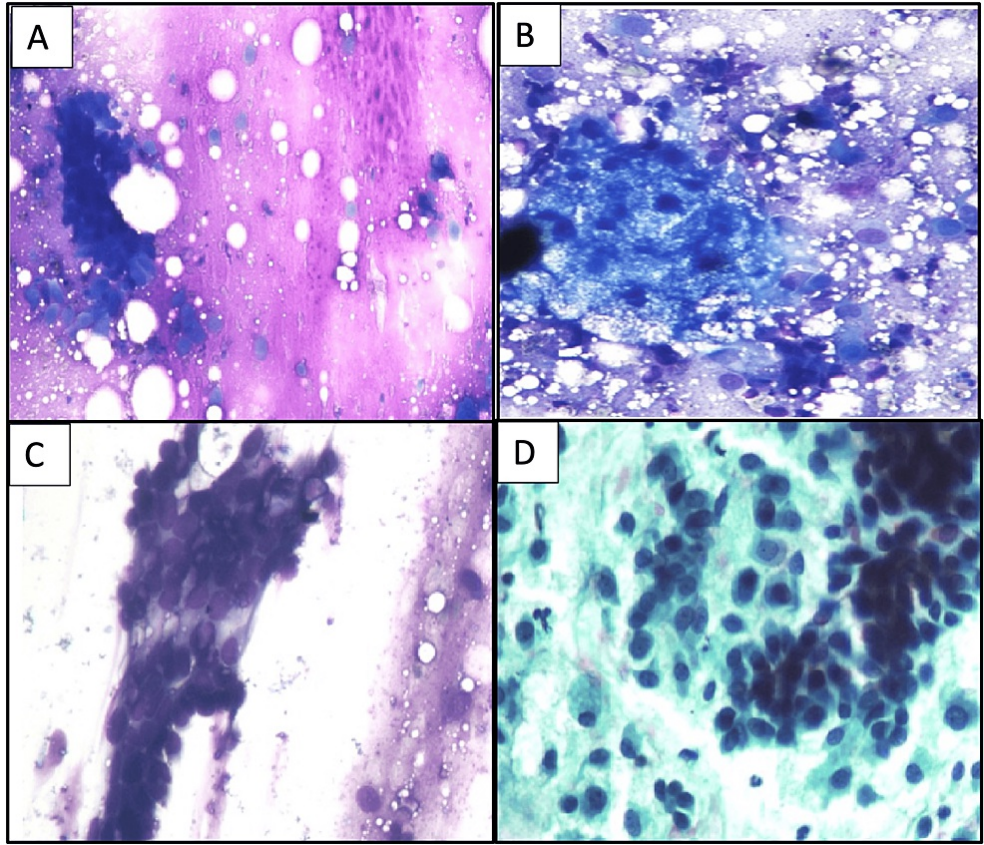
Cytological features of cases 1-4 A: Epithelial cell clusters in an abundant chondromyxoid matrix (case 1) (MGG stain, 10×). B: Epithelial cell cluster with focal sebaceous differentiation and singly scattered plasmacytoid cells in a hemorrhagic background (case 3) (MGG stain, 20×) C: Epithelial cell clusters showing ill-formed tubules on aspirates (case 2) (MGG stain, 40×). D: Cellular aspirates are composed of bland, monomorphic cells lying singly scattered and in clusters (case 4) (Papanicolaou stain, 40×). MGG: May Grunwald-Giemsa

FNAC from three of four cases aspirated thick mucoid material, and one yielded thin, clear, slightly mucoid, fluid-like material on aspiration. Figure 2 highlights the cytological features of CMT. All four aspirates were moderate to highly cellular with epithelial and stromal components. The cellular component comprised both epithelial and myoepithelial cells in clusters as well as singly scattered in a chondromyxoid background. The epithelial cells forming clusters were small, round to oval cells with a moderate amount of cytoplasm, bland, centrally located nuclei, and fine chromatin. The myoepithelial cells that were predominantly singly scattered were plasmacytoid to round to oval with a moderate amount of eosinophilic to amphophilic cytoplasm, eccentrically to centrally placed nuclei, bland chromatin, and inconspicuous nucleoli. These cells were found embedded in the fibrillary chondromyxoid stromal matrix. Although this background of stromal matrix, which is a typical feature of CMT, was predominantly myxoid and lacked chondroid differentiation in all our cases, the myxoid material (magenta purple) and its fibrillary nature were highlighted well on Giemsa-stained slides.

One of the four cases also showed the presence of large, round to oval cells with predominantly foamy cytoplasm and centrally located bland nuclei, suggestive of sebaceous differentiation (case 1). This was explained as a part of the metaplastic change often found in these tumors. However, no squamous differentiation or any other metaplastic element apart from a few clusters of sebaceous cells were seen in any of the cases aspirated.

Histological features

**Figure 3:**
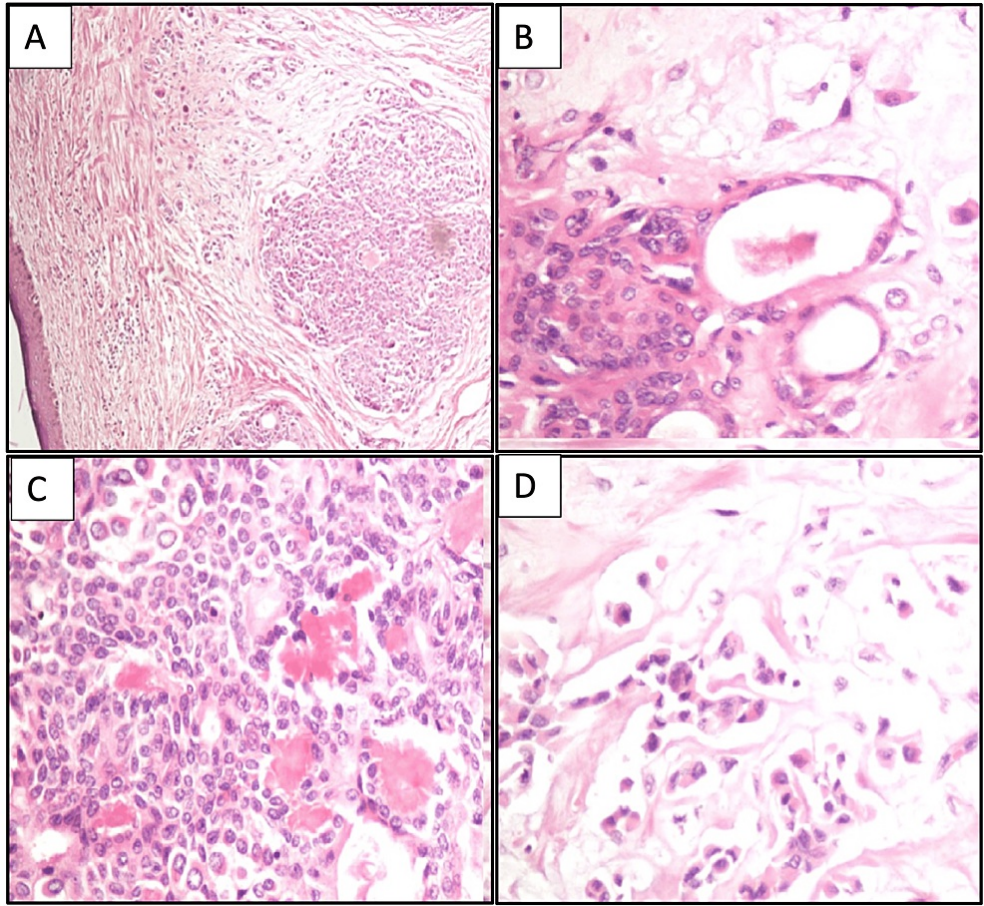
Histopathological features of cases 1-4 A and B: Histological section showing a tumor in the form of nests in the chondromyxoid matrix (cases 1 and 2) (hematoxylin and eosin, 20× and 40×). C: Epithelial cells arranged as nests, forming tubules and ducts at places (case 3) (hematoxylin and eosin, 40×). D: Singly scattered plasmacytoid cells in a chondromyxoid matrix (case 4) (hematoxylin and eosin, 40×).

The tumor was well circumscribed grossly in all cases. It was predominantly subepidermal, and microscopically, all of them had an epithelial component and a myoepithelial component to it. The epithelial component was forming nests, cords, tubules, and ducts, lined by myoepithelial cells in an abundant chondromyxoid matrix. These ducts had an inner cuboidal epithelial cell layer and an outer cell layer formed by myoepithelial cells. Singly scattered plasmacytoid myoepithelial cells were also seen at places in the chondromyxoid matrix. True chondroid differentiation was seen in one case. Spindled myoepithelial cells or squamous differentiation was not seen in any of the cases. Unfortunately, in our only case where sebaceous differentiation was seen on FNAC smears, multiple serial sections for histopathology failed to reveal the same (case 1). However, this could possibly be explained by the possible loss of tissue while trimming the sections in histopathology. Figure 3 highlights the histopathological features of cases 1-4.

Immunohistochemical features

**Figure 4:**
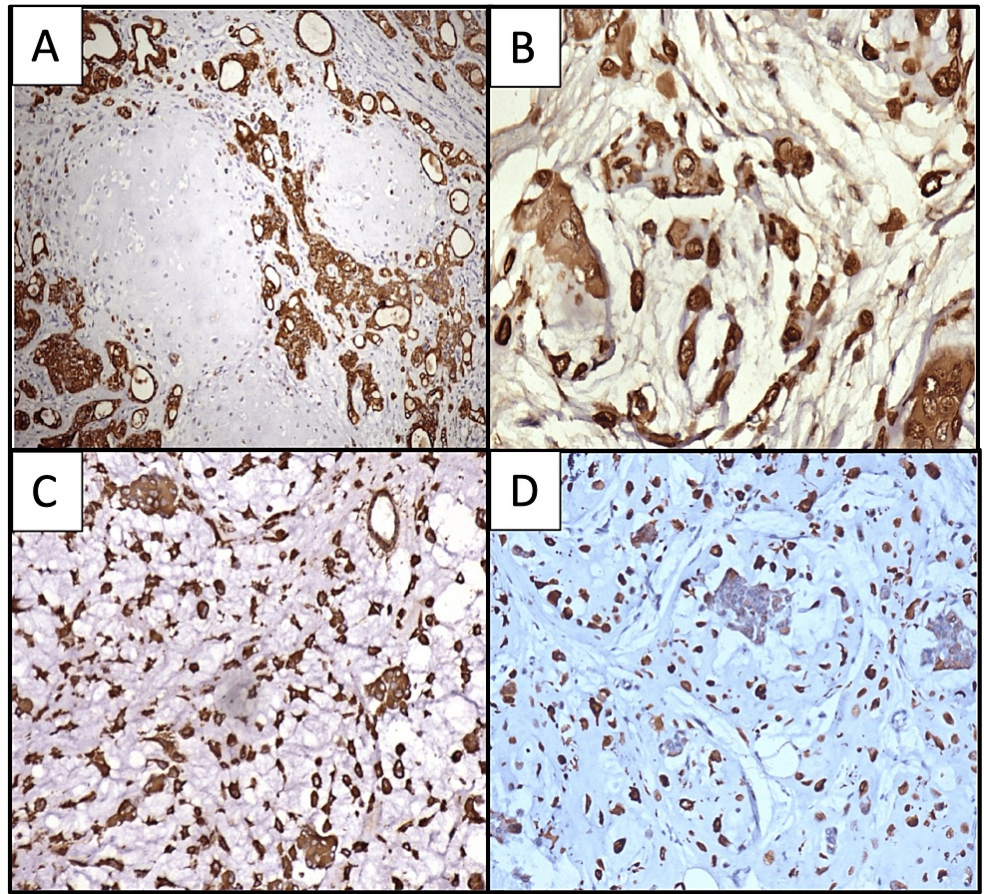
Immunohistochemical findings in cases 1-4 A: Both the inner and outer lining cells of the tubules and nests are cytokeratin positive (case 1) (cytokeratin, 10×). B: The outer lining cells (myoepithelial cells) and the stromal cells are immunopositive for S-100 (case 3) (S-100, 20×). C: Both myoepithelial and stromal cells are vimentin positive (case 4) (vimentin, 20×). D: Both myoepithelial and stromal cells are positive for GFAP (case 2) (GFAP, 20×). GFAP: glial fibrillary acidic protein

It was seen that while both the inner and outer cell layers were positive for pancytokeratin (PanCk), the outer cell layer and the singly scattered cells in the myxoid background were also positive for S-100, vimentin, and glial fibrillary acidic protein (GFAP). It was noted that GFAP was negative in the epithelial component. Figure 4 highlights the immunohistochemical findings in cases 1-4.

Table 2 summarizes the cytological and histopathological findings in cases 1-4.

**Table 2:** Summary of cytological and histopathological findings in cases 1-4 IHC: immunohistochemistry, PanCk: pancytokeratin, GFAP: glial fibrillary acid protein

Case number	Age/sex	Cytology findings	Cytological diagnosis	Microscopy (histological) findings	IHC for epithelial component	IHC for myoepithelial and stromal cells	Final histopathological diagnosis
1	25/male	Epithelial clusters in a chondromyxoid background	Cutaneous mixed tumors	Well-circumscribed, epithelial nests in a chondromyxoid background	Epithelial tubules positive for PanCk	Both myoepithelial and stromal cells are positive for vimentin, S-100, and GFAP	Cutaneous mixed tumor
2	45/male	Ill-formed epithelial tubules in a chondromyxoid background	Cutaneous mixed tumors	-do-	-do-	-do-	Cutaneous mixed tumors
3	35/female	Focal sebaceous differentiation and single plasmacytoid cells	Cutaneous mixed tumors	Epithelial nests and tubules in the matrix: sebaceous component not found	-do-	-do-	Cutaneous mixed tumors
4	55/female	Single and in clusters, bland monomorphic cells in a chondromyxoid background	Cutaneous mixed tumors	Singly scattered plasmacytoid cells in a chondromyxoid matrix	-do-	-do-	Cutaneous mixed tumors

## Discussion

Cutaneous mixed tumors or chondroid syringoma are skin adnexal tumors of sweat gland origin [1]. Accounting for up to 0.1 % of all adnexal tumors, these tumors are frequently benign but may be malignant in yet rarer cases. They are commonly seen in the head and neck regions, especially involving the nose, cheek, upper lip, scalp, forehead, and chin, in decreasing order of frequency [2]. Few other infrequent sites such as the axilla, thigh, trunk, and extremities have also been reported in the past [1-6]. While three of our cases were at commonly encountered sites, i.e., the cheek and forehead, one of our cases was at a relatively uncommon location involving the dorsal aspect of the right forearm. There are only a few case series correlating these tumors' cytomorphological and immunohistochemical features. Moreover, with the advent of newer molecular techniques, a number of newer genetic rearrangements have been reported in these tumors [7-9].

Since these tumors present as slow-growing nodular lesions of the skin, differential diagnoses of epidermal cyst, dermoid cyst, neurofibroma, dermatofibroma, pilomatrixoma, cutaneous histiocytoma, and rarely pleomorphic adenoma (depending on the location) are considered clinically [2]. In our study, two of four cases were clinically misdiagnosed as epidermal cysts, and only one of four was diagnosed as a skin adnexal tumor. On FNAC, the aspirates of these tumors demonstrated a biphasic cell population of both epithelial and myoepithelial cells in a predominantly fibrillary chondromyxoid matrix. The cytological findings in all our cases correlated well with the previously described studies. Plasmacytoid myoepithelial cells were seen in all four cases. The background matrix of CMT has been traditionally described as either myxoid, chondroid, chondromyxoid, or myxoid and chondroid [5,6]. Although true chondroid differentiation on cytology was not seen in any of our cases, a predominantly chondromyxoid matrix was present in all.

Histologically, there are two types of CMT: apocrine and eccrine [10]. These are multiphenotypic epithelial, myoepithelial, and mesenchymal neoplasm and thus can show many morphological patterns in cytology and histology [11]. While the apocrine types have a predominantly tubulocystic pattern with double cell layers, the eccrine types consist of a single layer of cuboidal epithelial cells surrounded by a chondromyxoid matrix. In all our four cases, there were tubules, nests, and islands of epithelial cells with intervening chondromyxoid stroma having plasmacytoid myoepithelial cells. Although one of our cases had chondroid differentiation on histopathology sections, it was not seen on cytology smears of the same.

Argenyi et al. studied the histogenesis of these tumors on immunohistochemistry and opined that these tumors are predominantly composed of tubuloglandular patterns with an inner and outer cell layer [12]. They concluded that the co-expression of cytokeratin (CK), vimentin, glial fibrillary acidic protein (GFAP), and S-100 in the outer myoepithelial cell layers and the stromal cells suggests the possible production of this mesenchymal component from outer cells. Our study also supports this hypothesis as all our cases were positive for CK in the inner layers of tubules and solid nests, while the singly scattered stromal cells and the outer cell layers were positive for CK, S-100, vimentin, and GFAP [12].

Atypical CMTs are cases with benign cytological features but with infiltrative margins and/or satellite nodules [13]. Malignant transformation in CMTs is indicated by the presence of cytological atypia, infiltrative margins, satellite tumor nodules, tumor necrosis, and involvement of deep structures [14]. Although these features may be difficult to appreciate in cytology alone, none of our four cases had these features in histology. In such cases, a definite clinicoradiological correlation may prove helpful in identifying the margins of the lesion, its expansive growth pattern, or the presence of lymphovascular invasion. Cell blocks may also play an important role in such cases.

In 2021, Macagno et al. reviewed the immunohistochemical and molecular features of CMTs. A panel of newer IHC markers such as SOX10, p63, PS-100, calponin, SMA, and desmin has been suggested to identify cells of multiple lineages in difficult cases [9]. This can also be useful when a cell block is available in the case of FNAC for performing IHC. Of these, SOX10 has not been studied in CS but is highly sensitive to pleomorphic adenoma of the salivary gland, although it lacks specificity. In some cases where there is follicular differentiation, IHC for BerEP4 and PHLDA1 can be used for confirmation.

A more specific marker called pleomorphic adenoma gene 1 (PLAG1) (clone 3B7) has recently been reported to be overexpressed in 87%-100% of CMTs. High-mobility group A2 (HMGA2) is another antibody that is being studied in a few cases of CMT. Unlike GFAP, PLAG1 is not seen in pure myoepithelioma that lacks ductal structures. However, due to the lower incidence of these tumors, studies conducted in this regard are still limited. Recurrent PLAG1 rearrangement has been found in approximately 33% of cases by fluorescence in situ hybridization (FISH) analysis. In 2020, Russell-Goldman et al. demonstrated that PLAG1 is differentially expressed in apocrine and eccrine types of CMT. Their results did not show any PLAG1 immunopositivity in eccrine CMT [11].

PLAG1 is a developmentally regulated zinc finger proto-oncogene that is activated through gene fusion with various partner genes such as CTNNB1, LIFR, and CHCHD7. It has been noted that this fusion typically occurs due to promoter swapping from various fusion partner genes that are ubiquitously expressed [9,11]. Unlike salivary gland tumors (pleomorphic adenoma) where multiple fusion partners have been identified for PLAG1, only two PLAG1 rearrangements (NDRG1:PLAG1 and TRPS:PLAG1 fusion) and no HMGA2 fusion have been identified in CMT so far. Flucke et al. identified Ewing sarcoma region 1 gene (EWSR1) rearrangement in a small subset of mixed tumors and myoepithelial carcinoma of the skin [15]. In 2019, Panagopoulos et al. reported PHF1-TFE3 gene fusion malignant CMT [16], but later in 2022, Zengin et al. reported the absence of TFE3 immunoexpression in the whole spectrum of CMT, including benign, atypical, and malignant cases [7].

CMTs are best treated by surgical excision as done in all our cases [2,12]. Other modalities such as electrodesiccation, dermabrasion, and vaporization with argon or CO2 laser have also been used in the past. All our patients were doing well until six months of surgery and had no recurrence of this tumor so far.

The strength of our study was the complete correlation of benign CMTs with their histopathological features and their confirmation with immunohistochemical markers in four cases of CMT. However, we feel that the addition of cell block and immunohistochemical features of newer antibodies such as PLAG1, TFE3, or EWSR1, along with their molecular confirmation using a more advanced technique such as FISH, would have been more advantageous in these cases. Further studies with more number of patients must be conducted to establish the role of FNAC and cell block in cases of CMT.

## Conclusions

Since the characteristic cytological features of CMTs in the form of biphasic cell population in a chondromyxoid matrix correlate well with the histopathological and immunohistochemical features, FNAC may be used as the first preoperative diagnostic modality in all cases of skin/subcutaneous nodular lesions. Excision is essential for a definitive diagnosis and ruling out malignancy in these cases. It is advisable to include margins of normal tissue during excision to avoid local recurrence in all cases. The utility of newer immunohistochemical markers such as PLAG1, TFE3, and EWSR1 on cell block still remains unexplored.
